# Complementary diesel-fuel compositional profiling by one-dimensional and comprehensive two-dimensional GC-TOF/MS

**DOI:** 10.1038/s41598-026-54777-6

**Published:** 2026-05-29

**Authors:** Tomasz Zieliński, Joanna Szpotkowska, Joanna Rudnicka, Aleksandra Bogumiła Florkiewicz, Edyta Szyszko, Tomáš Kovalczuk, Kamila Grygiel, Paweł Pomastowski

**Affiliations:** 1 Centre for Modern Interdisciplinary Technologies, Institute for Advanced Studies, Nicolaus Copernicus University in Toruń, Toruń, 87-100 Poland; 2 Energy Efficiency Project Coordination Team, ORLEN S.A, Płock, 09-411 Poland; 3 Department of Animal Biotechnology and Genetics, Bydgoszcz University of Science and Technology, Bydgoszcz, 85-084 Poland; 4 LECO Poland Ltd., Tychy, 43-100 Poland; 5 LECO Instrumente Plzeň Spol. Ltd., 301 00, Pilsen, Czech Republic

**Keywords:** Diesel fuel, 1D GC-TOF/MS, GC×GC-TOF/MS, Feature occurrence, Chemometrics, Compositional profiling, Chemistry, Environmental sciences

## Abstract

One-dimensional gas chromatography coupled with time-of-flight mass spectrometry (1D GC-TOF/MS) and comprehensive two-dimensional gas chromatography coupled with TOF/MS (GC×GC-TOF/MS) were evaluated as complementary platforms for diesel-fuel compositional profiling. Across all samples, 1D GC-TOF/MS reported 1,850 library-annotated features (similarity score ≥ 700), whereas GC×GC-TOF/MS reported 27,146 deconvoluted features, comprising 8,108 annotated features and 19,038 unnamed Peak-ID features. Only 595 annotated features were common to both workflows, yet workflow-specific statistical evaluation and PCA of GC×GC feature-occurrence profiles consistently highlighted the same atypical samples. Broad chemical grouping of annotated and predicted GC×GC features showed that paraffinic and olefinic/naphthenic hydrocarbon families dominated most fuels. In contrast, sample 51 × 57, and to a lesser extent K8LZ1 depending on feature inclusion, exhibited redistributed class patterns and a higher proportion of oxygenated or FAME-associated features. Integrating chromatographic profiling with routine physicochemical descriptors indicated that meaningful compositional divergence may occur even within a narrow specification window. Overall, 1D GC-TOF/MS remains effective for robust comparative screening, while GC×GC-TOF/MS provides additional selectivity and peak capacity to interrogate compositionally congested diesel matrices and to support future chemometric modelling.

**Supplementary Information:**

The online version contains supplementary material available at 10.1038/s41598-026-54777-6.

## Introduction

Diesel fuel remains a strategically important transport fuel, and its compositional quality directly influences ignition quality, combustion efficiency, exhaust emissions, storage stability and compliance with increasingly stringent engine-performance and environmental requirements^1–3^. At the molecular level, diesel is not a single substance but a compositionally dense petroleum-derived mixture containing alkanes, cycloalkanes, aromatic hydrocarbons and oxygenated components derived from additives, blending streams or degradation pathways. Reliable characterisation of this compositional landscape is therefore essential for refinery control, product authentication, contaminant tracing and interpretation of physicochemical fuel behaviour^4,5^.

Gas chromatography-mass spectrometry remains central to such analyses because it combines chromatographic separation with compound-specific spectral recognition, thereby enabling both targeted determination of known analytes and comparative fingerprinting of complex fuel matrices^6,7^. In routine petrochemical laboratories, one-dimensional GC continues to function as the practical gold standard because it is robust, comparatively easy to operate and well suited to high-throughput comparative screening. Its resolving power, however, becomes limiting when samples contain extended homologous series, numerous structural isomers and unresolved complex mixture regions, all of which are characteristic of middle distillates such as diesel fuel^8–10^.

Comprehensive two-dimensional gas chromatography (GC×GC) was developed precisely to address this analytical congestion. By coupling two columns with different selectivities through a modulation interface, GC×GC markedly increases peak capacity and creates a structured separation space in which chemically related subclasses occupy characteristic chromatographic zones^9,11^. For petrochemical samples, this added resolving power is particularly valuable because it improves discrimination of paraffins, cycloalkanes, olefins and aromatic subclasses that are often only partially separated in one-dimensional workflows^12–14^. In GC×GC, practical selectivity arises not only from the use of two stationary phases but also from modulation, which periodically traps, focuses and reinjects first-dimension effluent into the second-dimension column. This process converts unresolved or partially resolved first-dimension envelopes into a two-dimensional retention plane in which volatility-driven and selectivity-driven trends can be evaluated simultaneously. For diesel-range unresolved complex mixtures, such orthogonal ordering is particularly important because homologues, structural isomers and alkylated aromatics can otherwise be collapsed into composite one-dimensional peaks and mixed EI spectra^9,10,12,15–17^.

From a fuel-quality perspective, these class-level distinctions are chemically meaningful rather than merely descriptive. Paraffinic and isoparaffinic distributions influence ignition behaviour and cold-flow properties; aromatic enrichment is associated with soot-forming tendency and toxicological concern; sulfur- and nitrogen-containing species affect emissions, corrosion, catalyst compatibility and storage behaviour; and oxygenated components such as fatty acid methyl esters (FAMEs) modulate oxidation stability and deposit formation^18–22^. Consequently, the analytical question is not simply how many peaks can be detected, but how faithfully a given platform reconstructs the chemically relevant architecture of the sample. This distinction is also relevant for modern low-sulfur diesel fuels containing regulated FAME levels, because bulk quality parameters may remain within specification while subgroup distributions change in ways that affect oxidative stability, deposit formation, storage behaviour or source attribution. Thus, a workflow that preserves class-level structure can provide decision-relevant information beyond a simple pass/fail interpretation of density, sulfur, flash point or total FAME content^21–24^. Diesel-focused comparisons of GC-VUV, GC-FID and GC×GC-MS have similarly shown that complementary chromatographic and spectral workflows can provide mutually informative views of diesel and weathered-diesel composition^25,26^.

Against this background, the present study compares two analytical workflows for diesel‑fuel profiling: conventional 1D GC-TOF/MS and comprehensive two‑dimensional GC×GC-TOF/MS. We assess the extent to which the workflows agree at the level of library‑annotated features, and we examine how the substantially larger GC×GC feature space (including unnamed features) affects downstream chemometric interpretation. Specifically, we (i) quantify the overlap and asymmetry of annotated features between 1D and GC×GC workflows, (ii) evaluate within‑workflow sample similarity using correlation analysis, (iii) summarise GC×GC‑resolved composition using broad chemical families derived from annotation output, (iv) test the robustness of multivariate sample separation by performing PCA on annotated‑only and full GC×GC feature sets (including unnamed Peak IDs), and (v) relate chromatographic patterns to routine physicochemical descriptors used in fuel quality assessment.

## Materials and methods

### Material source

A coded set of diesel-fuel samples was analysed. Owing to commercial confidentiality, the provenance of the samples is not disclosed. The analytical dataset includes coded profiles that, in selected cases, are reported as lower, mixed or upper fractions of the same parent sample; these labels were retained exactly as generated during instrumental data processing to preserve traceability between figures and processed datasets.

Selected physicochemical properties were determined by the ORLEN Laboratory in Płock, and the results are summarised in Supplementary Table S1. The cetane index was determined according to PN-EN ISO 4264:2018-08, the cold filter plugging point according to PN-EN 116:2015-09 and the flash point (closed cup) according to PN-EN ISO 2719/PN-EN ISO 3015 procedures, as reported by the laboratory. Additional measurements included FAME content (PN-EN 14078:2014-06), sulfur content (PN-EN ISO 20846:2020-03) and contamination level (PN-EN 12662:2014-05).

### 1D GC-TOF/MS

Measurements were performed using an Agilent 6890 N gas chromatograph coupled to a LECO time‑of‑flight mass spectrometer. Separation was achieved on an Equity‑1 capillary column (30 m × 0.25 mm i.d. × 0.25 μm film; Supelco), which is a non‑polar 100% dimethylpolysiloxane stationary phase. The oven program was 40 °C for 3 min, then 20 °C min − 1 to 300 °C, followed by a 10 min hold to promote elution of late‑boiling diesel constituents. The injector temperature was 280 °C. Electron ionisation was performed at 70 eV in full‑scan mode over m/z 35–550. The ion‑source and transfer‑line temperatures were 225 and 280 °C, respectively. Complete transfer/elution under these conditions was verified by the absence of late‑eluting peaks in post‑run baselines and by lack of carry‑over in blank injections. Chromatographic data were acquired and processed using ChromaTOF software.

Each sample was analysed in triplicate. Tentative identifications were obtained by library matching against NIST, and features with a similarity score of at least 700 were retained as annotated features, whereas lower‑scoring matches were excluded from further interpretation. Triplicate peak lists were used to build a consensus profile per sample (presence/absence), thereby avoiding pseudo‑replication in downstream chemometric analyses. Throughout this study, the term “feature” denotes a deconvoluted chromatographic peak; “annotated feature” denotes a feature with a tentative library assignment rather than an independently confirmed structure, an interpretive distinction that is particularly critical for dense GC×GC data spaces.

### GC×GC-TOF/MS

GC×GC-TOF/MS analyses were performed on a platform comprising an Agilent 8890 gas chromatograph equipped with a split/splitless injector and a dual‑stage cryogenic modulator, coupled to a LECO TOF mass spectrometer. First‑dimension separation was performed on an Rxi‑17Sil MS column (30 m × 0.25 mm i.d. × 0.25 μm film; Restek), and second‑dimension separation on an Rxi‑5MS column (1.4 m × 0.25 mm i.d. × 0.25 μm film; Restek). Helium (> 99.999%) was used as the carrier gas at a constant flow rate of 1.0 mL min − 1. The primary oven program was 50 °C for 0.1 min, then 2.5 °C min − 1 to 330 °C, followed by a 1 min hold. The secondary oven was maintained 5 °C above the primary oven, and the modulator temperature was set 15 °C above the secondary oven. The modulation period was 5.0 s, the injector temperature 300 °C and the transfer‑line temperature 345 °C. GC×GC peak finding, spectral deconvolution and tentative annotation were performed in ChromaTOF using NIST library matching; features with a similarity score ≥ 700 were retained as annotated features, whereas lower-scoring peaks were exported as unnamed Peak‑ID features.

To maximise orthogonality while maintaining robust modulation of diesel‑range constituents, a moderately polar Rxi‑17Sil MS phase (50% phenyl/50% dimethyl polysiloxane) was used in the first dimension and a low‑polarity Rxi‑5MS phase (5% phenyl/95% dimethyl) in the second dimension, yielding a structured GC×GC retention space that supports group‑type interpretation of saturated, cyclic/unsaturated and aromatic families. The modulation period (tM = 5.0 s) was selected as a compromise between adequate reconstruction of first‑dimension peaks (typically providing ≥ 3 modulations per peak under the applied 2.5 °C min − 1 temperature program) and minimising second‑dimension wrap‑around, while ensuring stable cryogenic trapping and release in the dual‑stage modulator. The column configuration should therefore be interpreted as a class-structuring strategy rather than as a source of definitive compound identification: retention-space position supports group-type assignment, whereas structural confirmation would require retention indices, authentic standards and/or targeted MS evidence.

### Data processing and statistical analysis

Data processing and statistical analysis were carried out in Python using standard scientific libraries (pandas, numpy, scikit-learn and matplotlib). Peak-list exports (feature identifiers, library annotations, class labels and, where applicable, processed GC×GC descriptors) were converted into sample-by-feature occurrence matrices for chemometric analysis and visualisation. For group-type analyses, individual library-derived class labels were mapped to consolidated chemical families (Paraffins, Olefins/Naphthenes, Monoaromatics, Polyaromatics, Oxygenates and N-containing compounds) using a curated, structure-aware rule set based on functional groups and ring count. For the exploratory classification of unnamed Peak-ID features, first-dimension retention time, second-dimension retention time, base mass, peak signal-to-noise, quantification signal-to-noise and peak area were treated as multivariate descriptors; predicted labels were used only for class-level sensitivity analysis and were not considered confirmed identifications.

Pearson correlation analysis was used as an exploratory within-workflow similarity check based on binary feature-occurrence profiles. For GC×GC, PCA was performed on two matrices: annotated features only and the full feature set including unnamed Peak IDs. Prior to PCA, each matrix was mean-centred and decomposed by singular value decomposition (scikit-learn implementation). Importantly, PCA inputs were consensus sample profiles constructed after triplicate-level occurrence consolidation; individual technical replicates were not treated as independent observations. For categorical variables, statistical significance was evaluated with Pearson’s chi-square test. Distribution normality for quantitative variables was assessed using the Shapiro-Wilk test; non-normally distributed data were analysed using the Kruskal-Wallis test, whereas normally distributed data were evaluated using one-way ANOVA. Statistical significance was accepted at *p* < 0.05. Because 1D and GC×GC workflows differ in chromatographic configuration and feature definition, cross-workflow comparisons were interpreted at the level of profiling performance, class representation and multivariate structure rather than as direct one-to-one quantification of identical peaks. Representative 1D TIC chromatograms are provided in Supplementary Fig. S2, while GC×GC TIC contour plots and an example of EI mass-spectral deconvolution are shown in Supplementary Fig. S3.

## Results

### Feature overlap and annotation depth: 1D GC-TOF/MS versus GC×GC-TOF/MS

The two analytical workflows yielded markedly different feature counts. The 1D GC-TOF/MS workflow reported 1,850 library-annotated features (similarity score ≥ 700), whereas the GC×GC-TOF/MS workflow reported 27,146 deconvoluted features in total (including 8,108 library-annotated features and 19,038 unnamed features reported only as Peak IDs; Table 1). Overlap can be assessed only for annotated features: 595 annotated features were shared between workflows, corresponding to 32.2% of the 1D annotated list but only 7.3% of the GC×GC annotated list. This pronounced asymmetry is consistent with the larger chromatographic separation space and deconvolution capacity of comprehensive two‑dimensional GC, which resolves many more features than 1D GC under comparable library‑matching criteria^9–11^. Importantly, the modest overlap is not unexpected: extensive co‑elution in 1D GC yields mixed EI spectra that can return different (and occasionally spurious) library hits compared with the cleaner spectra obtained after comprehensive separation in GC×GC.

From a chromatographic perspective, the ca. 14.7-fold increase in the number of reported features in the GC×GC workflow should be interpreted primarily as an expansion of separation space rather than as a proportional increase in structurally confirmed compounds. In diesel-range matrices, orthogonal separations resolve homologous and isomeric envelopes that remain partially collapsed in one-dimensional GC, and the resulting overlap asymmetry shows that the two workflows do not simply report the same chemistry with different sensitivity. Instead, they reconstruct partially different analytical views of the same fuel matrix because peak capacity, co-elution behaviour and spectral deconvolution differ across platforms^23,24,26–29^. These differences are illustrated by representative 1D TIC chromatograms in Supplementary Fig. S2 and by GC×GC contour and deconvolution/mass-spectral examples in Supplementary Fig. S3.

**Table 1:** Summary of reported features obtained by 1D GC-TOF/MS and GC×GC-TOF/MS across the full set of diesel samples. Percentages for annotated and unnamed features are calculated relative to the total number of reported GC×GC features. Percentages for shared/unique features are calculated relative to the number of annotated features in the corresponding workflow.

Metric	Workflow	Value
Total reported features	1D GC-TOF/MS	1,850 (100.0% annotated)
Total reported features	GC×GC-TOF/MS	27,146
Annotated features (library-matched; similarity score ≥ 700)	1D GC-TOF/MS	1,850 (100.0% of total features)
Annotated features (library-matched; similarity score ≥ 700)	GC×GC-TOF/MS	8,108 (29.9% of total features)
Unnamed features (Peak ID only; no library name)	GC×GC-TOF/MS	19,038 (70.1% of total features)
Shared annotated features (intersection between workflows)	Both	595 (32.2% of 1D annotated; 7.3% of GC×GC annotated)
Unique annotated features	1D GC-TOF/MS	1,255 (67.8% of 1D annotated)
Unique annotated features	GC×GC-TOF/MS	7,513 (92.7% of GC×GC annotated)

To complement the feature-count comparison, class-level occurrence patterns were evaluated by Pearson’s chi-square tests for each workflow (Supplementary Fig. S1). The GC×GC-TOF/MS panel displays a denser set of low p-values across aromatic and FAME-linked categories than the 1D GC-TOF/MS panel, indicating that the comprehensive separation produces more statistically differentiated class-occurrence patterns within the studied sample set. These tests were used as exploratory descriptors of between-sample differentiation and not as evidence of absolute concentration differences.

The statistical comparison in Supplementary Fig. S1 is consistent with the broader interpretation that GC×GC-TOF/MS reveals a richer and more differentiated class space than 1D GC-TOF/MS. Importantly, this additional chromatographic structure is observed in fuels whose routine physicochemical descriptors occupy a narrow range (Supplementary Table S1), particularly for density, cetane index, FAME content and sulfur. Thus, chromatographic profiling reveals sub-specification-level redistribution of hydrocarbon subclasses and FAME-associated signals that would be difficult to infer from routine bulk measurements alone^12,13,18,24^.

### Chemical-class composition of annotated GC×GC features

Because the GC×GC export contained a large number of unnamed Peak-ID features, we performed an exploratory retention-space and spectral-descriptor classification to assess whether these features modified class-level trends. Uniform Manifold Approximation and Projection (UMAP) was used to project the multivariate descriptor space, and Hierarchical Density-Based Spatial Clustering of Applications with Noise (HDBSCAN) was used to define density-based clusters. Cluster labels were inferred by nearest-neighbour comparison with annotated features. The descriptor set included first- and second-dimension retention times, base mass, peak signal-to-noise, quantification signal-to-noise and peak area. These assignments should be regarded as predicted class labels rather than additional compound identifications^15,16,26^.

**Fig. 1:**
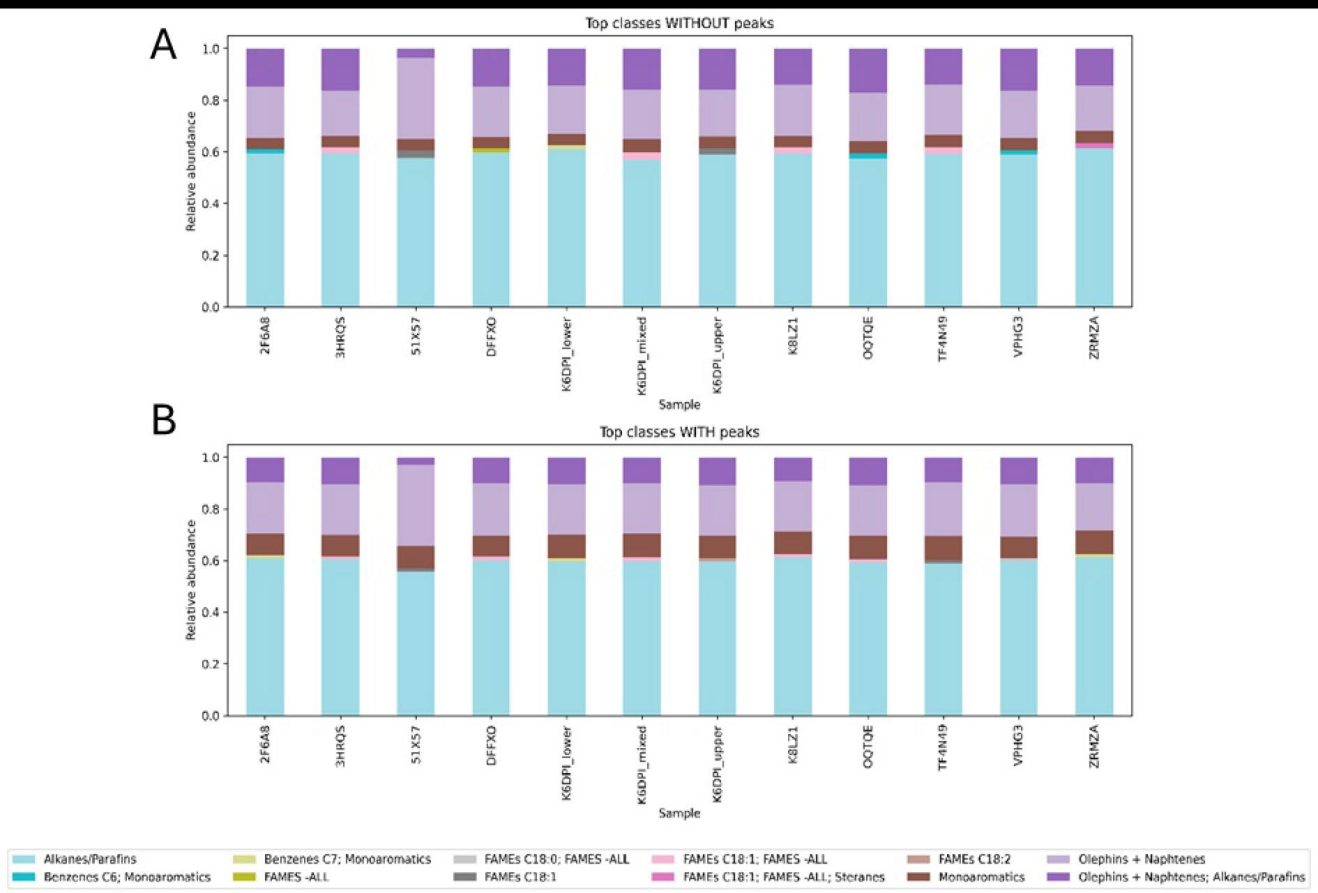
Stacked bar plots showing relative GC×GC-derived chemical-class composition across samples (**A**) after exclusion of predicted Peak-ID features and (**B**) after inclusion of predicted Peak-ID features. In the figure labels, “peaks” denotes unnamed Peak-ID features to which a broad predicted class was assigned by the exploratory UMAP/HDBSCAN-nearest-neighbour workflow.

Class distributions calculated without and with predicted Peak-ID features are shown in Fig. 1. Inclusion of these features increased monoaromatic representation consistently across the samples, but the overall class architecture and relative sample ordering were retained. This result supports the view that the unnamed GC×GC feature space is chemically structured rather than random analytical noise.

**Fig. 2:**
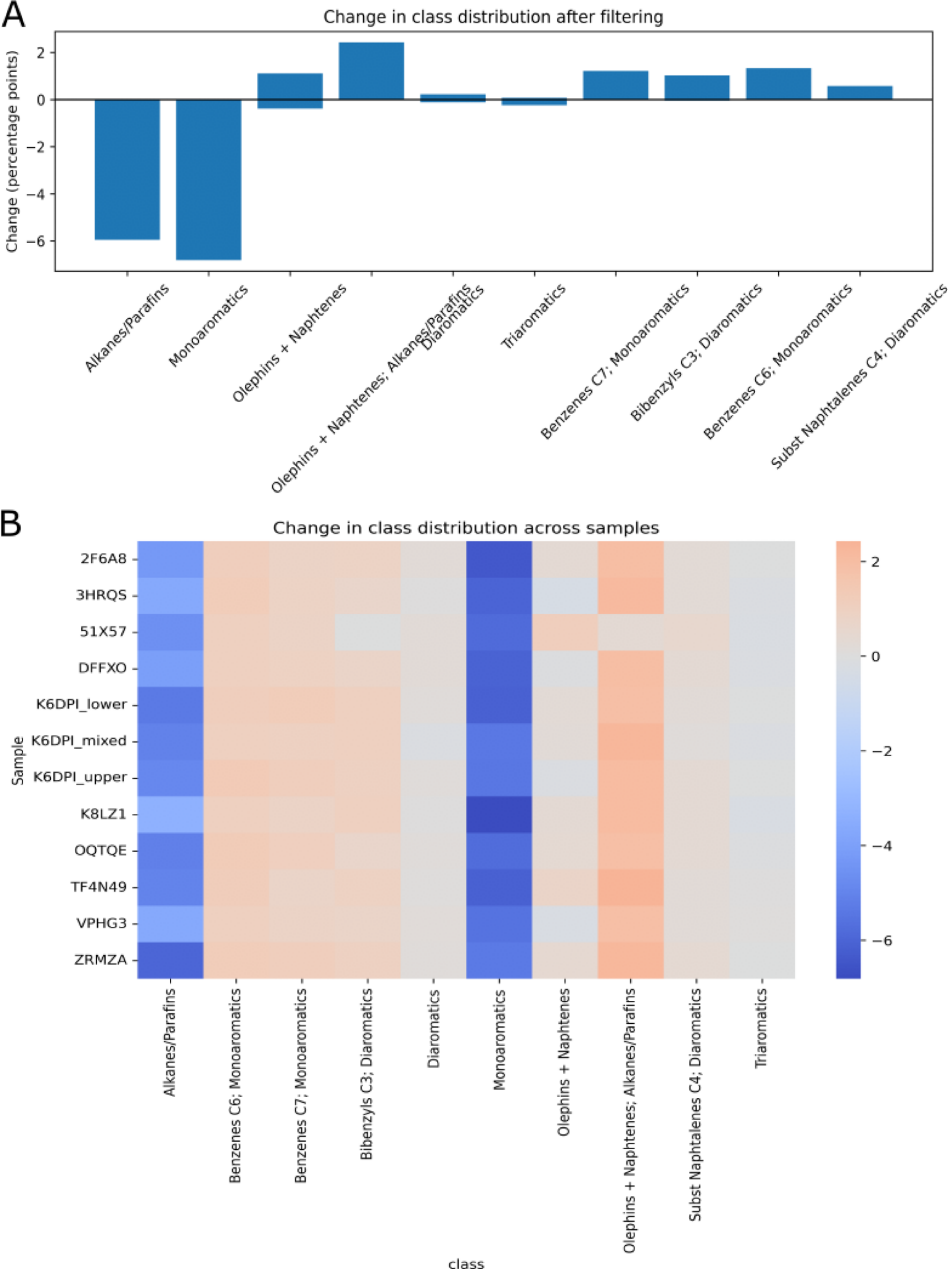
Changes in compound class distribution following removal of predicted peak features. (**A**) Overall class distribution across the dataset, showing a decrease in alkanes/paraffins and monoaromatics and minor changes in other classes. (**B**) Heatmap of class distributions across individual samples, indicating highly similar composition patterns with only small variations after peak removal. Predicted peak features denote unnamed Peak-ID features assigned to broad classes by the UMAP/HDBSCAN-nearest-neighbour workflow.

We further assessed how removal of predicted Peak-ID features affected class distribution across the entire dataset (Fig. 2A) and within individual samples (Fig. 2B). Removal resulted in an approximately 6% decrease in both alkanes/paraffins and monoaromatics, accompanied by only minor increases in the remaining classes. At the sample level, the maximum shift in class enrichment following feature removal did not exceed 2% points. Sample 51 × 57 retained its enrichment in olefinic/naphthenic features, indicating that the principal class-level conclusion does not depend on predicted assignments alone.

These findings strengthen the sensitivity analysis requested during review, but they also define its interpretive boundary: predicted labels are valuable for pattern confirmation and quality-screening hypotheses, whereas compound-level claims require retention-index support, authentic standards and/or targeted validation.

### Multivariate analysis of GC×GC feature-occurrence profiles

Principal component analysis (PCA) was applied to the GC×GC-TOF/MS dataset to evaluate sample relationships and to test whether the high proportion of unnamed (Peak‑ID) features affects multivariate structure. Two PCA models were constructed from binary feature‑occurrence matrices (presence/absence): (i) annotated features only (*n* = 8,108 variables) and (ii) the full feature set including unnamed Peak IDs (*n* = 27,146 variables).

**Fig. 3:**
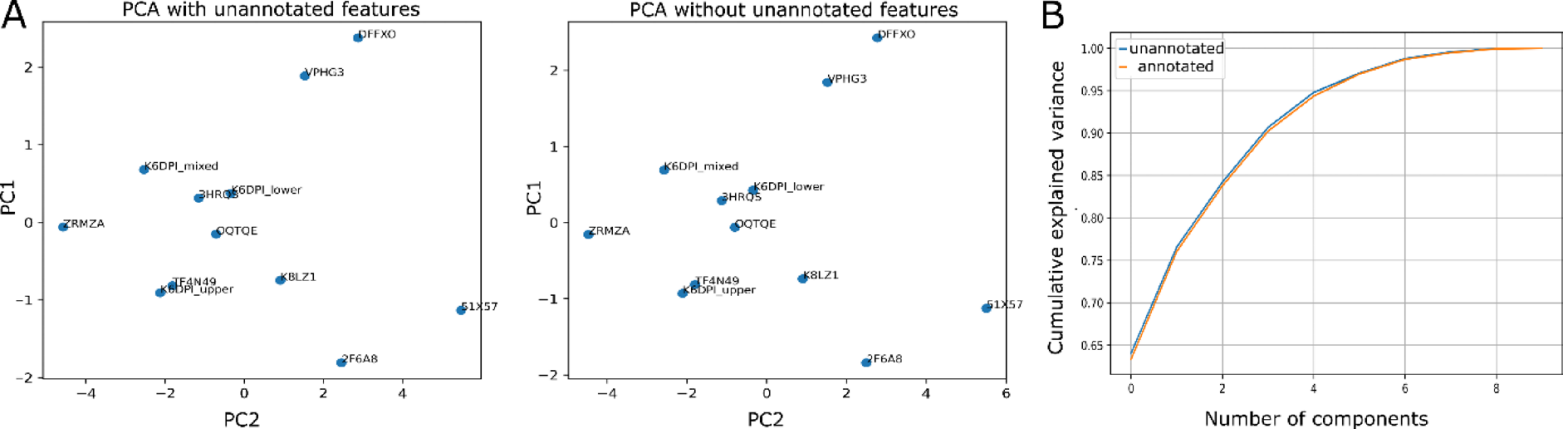
Principal component analysis (PCA) of GC×GC-TOF/MS feature-occurrence profiles. (**A**) Score plots for the full feature set including unnamed Peak-ID features and for annotated features only. (**B**) Cumulative explained variance as a function of the number of principal components for the corresponding datasets.

In both PCA models, 51 × 57 was strongly separated from the remaining fuels, confirming it as the dominant outlier (Fig. 3). In the full‑feature model, K8LZ1 also deviated from the central cluster, whereas the three K6DPI distillation fractions remained closely grouped, consistent with their common origin. The persistence of the same outliers when unnamed features were included indicates that Peak-ID features carry discriminatory information rather than acting as unstructured noise. This interpretation is further supported by the class-level sensitivity analysis in Figs. 1 and 2, where inclusion of predicted Peak-ID features changes absolute class proportions but not the principal sample-level pattern.

As expected for high‑dimensional binary occurrence data, the first two principal components each captured only a modest fraction of total variance (≈ 10–11%), yet they were sufficient to visualise robust sample‑level divergence.

## Discussion

Gas chromatography coupled with mass spectrometry is a cornerstone analytical strategy for petroleum characterisation because it enables simultaneous separation, tentative identification and comparative profiling of volatile and semi‑volatile constituents^6,7,9,11^. The present results show that, for diesel fuel, the decisive analytical question is not only how many features can be detected, but how effectively those features can be converted into chemically interpretable information. In that respect, the two workflows examined here produced complementary rather than interchangeable views of the same matrix. The historical development of GC×GC in petroleum analysis is instructive in this regard. Early studies by Venkatramani and Phillips and, later, by Frysinger and Gaines established that the principal value of comprehensive separations lies not merely in generating more peaks, but in disentangling structured hydrocarbon continua into interpretable chromatographic bands^8–11,30,31^. The present GC×GC-TOF/MS dataset conforms closely to that paradigm: the method expands the observable space of diesel composition and, more importantly, partitions that space into subclasses that can be interpreted in chemically meaningful terms.

The most immediate difference between the workflows was the much larger feature space produced by GC×GC-TOF/MS. This outcome is expected for highly complex petroleum matrices, in which one‑dimensional GC is prone to co‑elution and therefore compresses chemically distinct constituents into fewer composite features. Importantly, the larger number of resolved GC×GC features primarily reflects increased chromatographic peak capacity and more effective deconvolution, rather than necessarily superior absolute instrumental sensitivity; quantitative LOD/LOQ comparisons were outside the scope of this study.

At the same time, the GC×GC dataset illustrates a central limitation of deep untargeted profiling: detection depth increases more rapidly than confident annotation. This is consistent with the limited coverage of petroleum-derived constituents in general-purpose EI mass-spectral libraries and with the high spectral similarity among isomeric hydrocarbons. Even with a relatively stringent similarity threshold, more than 70% of GC×GC features were retained only as Peak IDs (Table 1). To evaluate whether these unnamed features should be treated as analytical “noise” or as potentially informative descriptors, we compared PCA based on annotated features with PCA based on the full feature set including Peak IDs (Fig. 3) and complemented this analysis with class-level sensitivity testing (Figs. 1 and 2). The persistence of the same dominant outliers across these comparisons suggests that unnamed features can capture real compositional structure, although interpretation requires additional annotation, retention-space clustering and/or validation strategies.

The dominance of paraffinic and olefinic/naphthenic hydrocarbon families among annotated GC×GC features (Figs. 1 and 2) is consistent with the expected compositional envelope of diesel fuel, which is governed by C10-C22 hydrocarbons with smaller contributions from aromatics and oxygenated components (including FAME in B7 fuels)^30,31^. Because our class summaries are occurrence‑based, they should be interpreted as reflecting feature richness rather than absolute abundance.

Sample‑specific behaviour further illustrates the chemical value of chromatographic profiling. Sample 51 × 57 reproducibly diverged from the remaining fuels in class composition (Fig. 2), within‑workflow correlation structure (Supplementary Fig. S1) and PCA (Fig. 3). This divergence was expressed as a reduced paraffinic fraction together with an increased proportion of unclassified and oxygenated annotated features, suggesting compositional differences not fully captured by routine bulk descriptors. K8LZ1 and VPHG3 also displayed workflow‑dependent deviations, despite remaining within specification for density, sulfur and flash point (Supplementary Table S1).

A further important observation is that multivariate outlier detection can be robust even when feature‑by‑feature overlap between workflows is modest. Although only 595 annotated features were common to both workflows (Table 1), the same samples (most notably 51 × 57) were consistently highlighted as atypical in independent similarity analyses. This indicates that higher‑order compositional patterns, rather than exact agreement in individual tentative identifications, can drive reproducible discrimination of fuel samples.

Several limitations should be acknowledged. First, the study relies on tentative library‑based assignments rather than systematic confirmation by retention indices and authentic standards. Second, the sample set represents a limited physicochemical envelope of commercial low‑sulfur B7 diesel fuels; therefore, the generality of the observed feature‑space relationships to other diesel types (e.g., higher‑sulfur fuels, different FAME blends or alternative feedstocks) remains to be established. Third, cross‑workflow quantitative comparisons are complicated by differences in modulation, peak shapes and deconvolution behaviour; consequently, our primary comparisons focus on feature richness and multivariate structure rather than absolute concentration.

Future work should build on this framework in two complementary directions. First, representative alkylbenzene, naphthenic and methyl‑ester markers should be anchored by retention indices and authentic standards, which would translate the present class‑level trends into firmer compound‑level statements. Second, the dataset al.ready points toward composition-property modelling. Recent studies have shown that class‑resolved chromatographic data can be used to predict density, lower heating value, combustor operability and related performance descriptors for complex fuels^32–41^. The present results are compatible with that strategy because the most robust information in these diesel samples resides at the class and multivariate levels rather than in exhaustive single‑compound enumeration.

From an analytical‑mechanistic perspective, selectivity, peak capacity and effective detectability should be distinguished carefully. The present study did not determine instrumental limits of detection or quantification experimentally, so any claim of superior absolute sensitivity would be unwarranted. What the data do support is improved effective detectability in the GC×GC workflow: by spreading chemically similar analytes over two retention dimensions, the method reduces co‑elution‑driven masking and makes low‑abundance aromatic or ester‑linked subclasses easier to discern within a complex background. In parallel, systematic exploitation of unnamed features via retention‑space binning and/or spectral‑similarity clustering could convert Peak‑ID features into reproducible descriptors even when full chemical annotation is not available. In practical non-targeted GC×GC workflows, unnamed features can also be exploited through pixel-based or tile-based analysis, in which the two-dimensional chromatogram is partitioned into retention-time bins or chemically informed regions and each pixel/tile is treated as a reproducible descriptor. Such approaches reduce dependence on complete library annotation, can tolerate small retention shifts after alignment and are compatible with PCA, PLS, Fisher-ratio screening or supervised classification models^15,16^. In the present study, the occurrence-based matrices and predicted Peak-ID classes represent a conservative implementation of this concept; future work should move toward aligned, intensity-aware pixel-level descriptors for composition-property modelling.

Conversely, the practical value of one‑dimensional workflows should not be understated. Standardised single‑dimension methods remain indispensable in routine control because they are robust, easier to transfer between laboratories and often sufficiently precise for major hydrocarbon classes. Recent fuel‑characterisation studies show that established routine methods can satisfy precision requirements for dominant classes, whereas comprehensive GC becomes most advantageous when low‑level classes, unusual matrices or strongly overlapping compositional domains must be resolved^17,4243,44,45,46,47,48,49^. The present dataset follows the same pattern: 1D GC-TOF/MS was capable of flagging the principal outlier, while GC×GC-TOF/MS supplied additional compositional context and a richer feature space for chemometric exploitation.

## Conclusions

GC×GC-TOF/MS provided a substantially deeper and more structurally resolved representation of diesel-fuel composition than 1D GC-TOF/MS, reflecting the higher peak capacity and improved co-elution mitigation of comprehensive two-dimensional separation. At the same time, routine library matching captured only a minority of the GC×GC feature space, emphasising that untargeted diesel profiling rapidly becomes annotation-limited. Despite this limitation, multivariate analyses based on feature-occurrence profiles were robust: key outliers persisted when the GC×GC dataset was analysed using annotated features only or the full feature set including unnamed Peak IDs. Together, these results support a tiered and complementary analytical strategy, in which 1D methods remain valuable for routine screening, while GC×GC adds depth for discriminating subtle compositional differences and enabling future chemometric or pixel-based models.

## Supplementary Information

Below is the link to the electronic supplementary material.

Supplementary Material 1

## Data Availability

The data supporting this article have been included as part of the Supplementary Information. Additional metadata concerning the origin of the samples are not publicly disclosed because of commercial confidentiality restrictions.
