# Supplementary material for: Complementary diesel-fuel compositional profiling by one-dimensional and comprehensive two-dimensional GC-TOF/MS
Source: Sci Rep. 2026 May 29;16:24634. doi: 10.1038/s41598-026-54777-6 (PMC13454619; doi:10.1038/s41598-026-54777-6)
Supplement: Supplementary file 1 [file 41598_2026_54777_MOESM1_ESM.pdf]

Supplementary Note. The bulk physicochemical descriptors occupy a relatively narrow range across the analysed fuels: density 836.3-839.3 kg m^{-3}, cetane index 51.6-52.4, total FAME content 6.7-7.2% (*v/v*) and sulfur 6.4-8.1 mg kg^{-1}. This restricted window provides an important external reference frame for the chromatographic results discussed in the main manuscript. In particular, it shows that the separation of 51X57, VPHG3, K8LZ1 and the K6DPI-derived profiles cannot be explained simply by gross off-specification behaviour. Instead, the 1D GC-TOF/MS and GC×GC-TOF/MS datasets resolve finer redistribution of hydrocarbon subclasses, aromatic signals and FAME-associated features that remain only weakly expressed in routine bulk descriptors. Supplementary Fig. S1 supports the class-occurrence statistical comparison in the Results; Supplementary Fig. S2 documents the 1D TIC-level contrast between a representative sample and the outlier; and Supplementary Fig. S3 provides the corresponding GC×GC contour and EI mass-spectral/deconvolution evidence for co-elution in the 51X57 profile.

Supplementary Table S1. Physicochemical properties of the analysed diesel fuel samples. "Distillation temperature at 95% recovery (T95)" is reported as a single characteristic point from the distillation curve. "Total contamination" corresponds to the insoluble particulate contamination measured by the standard method.

Sample	Type of fuel	Parameters	Unit	Value	Raw value	Temperature [°C]
0QTQE	VERVA ON	up to 250 °C distills	% (*v/v*)	34.1	34.1	-
2F6A8	EFECTA ON			34.3	34.3	-
3HRQS	EFECTA ON			36.2	36.2	-
51X57	EFECTA ON			34.6	34.6	-
DFFX0	EFECTA ON			35.5	35.5	-
K6DPI	VERVA ON			36	36	-
K8LZ1	VERVA ON			35.6	35.6	-
TF4N49	VERVA ON			35.3	35.3	-
VPHG3	VERVA ON			36.2	36.2	-
ZRMZA	EFECTA ON			34.2	34.2	-
0QTQE	VERVA ON	up to 350 °C distills	% (*v/v*)	93.8	93.8	-
2F6A8	EFECTA ON			94.4	94.4	-
3HRQS	EFECTA ON			93.3	93.3	-
51X57	EFECTA ON			93.5	93.5	-
DFFX0	EFECTA ON			92.8	92.8	-
K6DPI	VERVA ON			93.7	93.7	-
K8LZ1	VERVA ON			93.7	93.7	-
TF4N49	VERVA ON			93.9	93.9	-
VPHG3	VERVA ON			93.2	93.2	-
ZRMZA	EFECTA ON			94.1	94.1	-
0QTQE	VERVA ON	Density	kg/m³	838.7	838.7	15
2F6A8	EFECTA ON			839.3	839.3	15
3HRQS	EFECTA ON			836.4	836.35	15
51X57	EFECTA ON			837.9	837.9	15

DFFX0	EFECTA ON			836.3	836.3	15
K6DPI	VERVA ON			837.1	837.13	15
K8LZ1	VERVA ON			838.2	838.2	15
TF4N49	VERVA ON			838.1	838.1	15
VPHG3	VERVA ON			836.5	836.46	15
ZRMZA	EFECTA ON			839	839	15
0QTQE	VERVA ON	Cetane index	-	52.3	52.3	-
2F6A8	EFECTA ON			51.8	51.8	-
3HRQS	EFECTA ON			52.4	52.4	-
51X57	EFECTA ON			51.6	51.6	-
DFFX0	EFECTA ON			52.3	52.3	-
K6DPI	VERVA ON			52.3	52.3	-
K8LZ1	VERVA ON			52.1	52.1	-
TF4N49	VERVA ON			52.1	52.1	-
VPHG3	VERVA ON			52.1	52.1	-
ZRMZA	EFECTA ON			51.9	51.9	-
0QTQE	VERVA ON	Distillation temperature at 95% recovery (T95)	°C	354.3	354.3	-
2F6A8	EFECTA ON			351.7	351.7	-
3HRQS	EFECTA ON			355.2	355.2	-
51X57	EFECTA ON			355.7	355.7	-
DFFX0	EFECTA ON			357.3	357.3	-
K6DPI	VERVA ON			353.8	353.8	-
K8LZ1	VERVA ON			355.3	355.3	-
TF4N49	VERVA ON			354.7	354.7	-
VPHG3	VERVA ON			355.2	355.2	-
ZRMZA	EFECTA ON			353.3	353.3	-
0QTQE	VERVA ON	Cetane number derivative	-	55.6	55.6	-
K8LZ1	VERVA ON			56.5	56.5	-
VPHG3	VERVA ON			56.4	56.4	-
0QTQE	VERVA ON	Combustion delay (medium)	ms	4.28	4.28	-
K8LZ1	VERVA ON			4.22	4.22	-
VPHG3	VERVA ON			4.22	4.22	-
0QTQE	VERVA ON	Ignition delay (medium)	ms	2.74	2.74	-
K8LZ1	VERVA ON			2.69	2.69	-
VPHG3	VERVA ON			2.71	2.71	-
0QTQE	VERVA ON	Corrected average wear scar diameter	µm	210	210	60
K8LZ1	VERVA ON			200	200	60
VPHG3	VERVA ON			190	190	60
0QTQE	VERVA ON	Oxidation stability	h	50	62.1	110
2F6A8	EFECTA ON			50	66	110
3HRQS	EFECTA ON			48	48.5	110
51X57	EFECTA ON			50	75.1	110
DFFX0	EFECTA ON			48	48.5	110
K6DPI	VERVA ON			48	48.5	110
K8LZ1	VERVA ON			50	60.4	110
TF4N49	VERVA ON			50	67.8	110

VPHG3	VERVA ON			48	48.5	110
ZRMZA	EFECTA ON			50	73.8	110
0QTQE	VERVA ON		g/m³	6	6	-
K8LZ1	VERVA ON			4	4	-
VPHG3	VERVA ON			6	6	-
0QTQE	VERVA ON	Flash point closed cup	°C	62.5	62.5	-
2F6A8	EFECTA ON			63.5	63.5	-
3HRQS	EFECTA ON			62.5	62.5	-
51X57	EFECTA ON			61.5	61.5	-
DFFX0	EFECTA ON			60.5	60.5	-
K6DPI	VERVA ON			62.5	62.5	-
K8LZ1	VERVA ON			65.5	65.5	-
TF4N49	VERVA ON			63.5	63.5	-
VPHG3	VERVA ON			61.5	61.5	-
ZRMZA	EFECTA ON			64.5	64.5	-
0QTQE	VERVA ON	Appearance	-	bright, transparent, without visible impurities and water		-
2F6A8	EFECTA ON			bright, transparent, without visible impurities and water		-
3HRQS	EFECTA ON			bright, transparent, without visible impurities and water		-
51X57	EFECTA ON			bright, transparent, without visible impurities and water		-
DFFX0	EFECTA ON			bright, transparent, without visible impurities and water		-
K6DPI	VERVA ON			bright, transparent, without visible impurities and water		-
K8LZ1	VERVA ON			bright, transparent, without visible impurities and water		-
TF4N49	VERVA ON			bright, transparent, without visible impurities and water		-
VPHG3	VERVA ON			bright, transparent, without visible impurities and water		-
ZRMZA	EFECTA ON			bright, transparent, without visible impurities and water		-
0QTQE	VERVA ON	Fatty acid methyl esters (FAME) content	% (*v/v*)	7.2	7.2	-
2F6A8	EFECTA ON			7.2	7.2	-
3HRQS	EFECTA ON			6.8	6.8	-

51X57	EFECTA ON			7.1	7.1	-
DFFX0	EFECTA ON			6.7	6.7	-
K6DPI	VERVA ON			6.8	6.8	-
K8LZ1	VERVA ON			7	7	-
TF4N49	VERVA ON			7	7	-
VPHG3	VERVA ON			6.7	6.7	-
ZRMZA	EFECTA ON			7	7	-
0QTQE	VERVA ON	Total sulfur content	mg/kg	8.1	8.1	-
2F6A8	EFECTA ON			7.4	7.4	-
3HRQS	EFECTA ON			6.4	6.4	-
51X57	EFECTA ON			7.3	7.3	-
DFFX0	EFECTA ON			6.4	6.4	-
K6DPI	VERVA ON			7.1	7.1	-
K8LZ1	VERVA ON			6.9	6.9	-
TF4N49	VERVA ON			7.5	7.5	-
VPHG3	VERVA ON			6.7	6.7	-
ZRMZA	EFECTA ON			8	8	-
0QTQE	VERVA ON	Water content	% (*m/m*)	0.008	0.008	-
2F6A8	EFECTA ON			0.009	0.009	-
3HRQS	EFECTA ON			0.006	0.006	-
51X57	EFECTA ON			0.009	0.009	-
DFFX0	EFECTA ON			0.007	0.007	-
K6DPI	VERVA ON			0.007	0.007	-
K8LZ1	VERVA ON			0.009	0.009	-
TF4N49	VERVA ON			0.009	0.009	-
VPHG3	VERVA ON			0.006	0.006	-
ZRMZA	EFECTA ON			0.01	0.01	-
0QTQE	VERVA ON	Total contamination	mg/kg	6	5.5	-
2F6A8	EFECTA ON			7.5	7.5	-
3HRQS	EFECTA ON			12	4	-
51X57	EFECTA ON			6.5	6.5	-
DFFX0	EFECTA ON			12	8.5	-
K6DPI	VERVA ON			12	6.5	-
K8LZ1	VERVA ON			6.5	6.5	-
TF4N49	VERVA ON			20.5	20.5	-
VPHG3	VERVA ON			12	6	-
ZRMZA	EFECTA ON			10.5	10.5	-

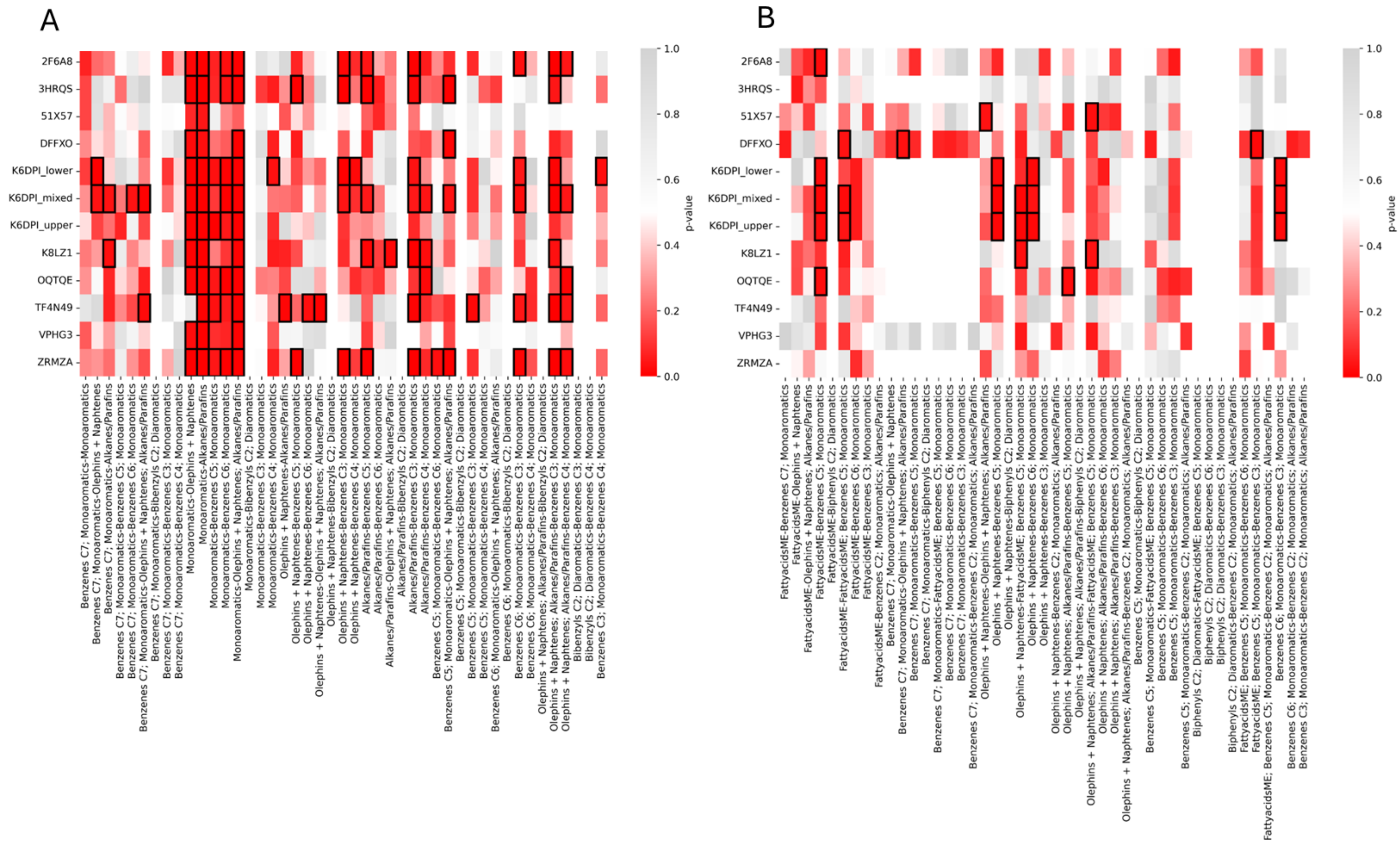

Supplementary Figure S1. Statistical comparison of class occurrence across samples for GC×GC-TOF/MS (A) and 1D GC-TOF/MS (B). Heatmaps show p-values from Pearson's chi-square tests; lower p-values (red) indicate stronger between-sample differentiation within a given class. The GC×GC panel resolves a denser set of differentiated aromatic and FAME-linked classes than the 1D panel, consistent with the higher peak capacity of the comprehensive workflow for compositionally congested samples.

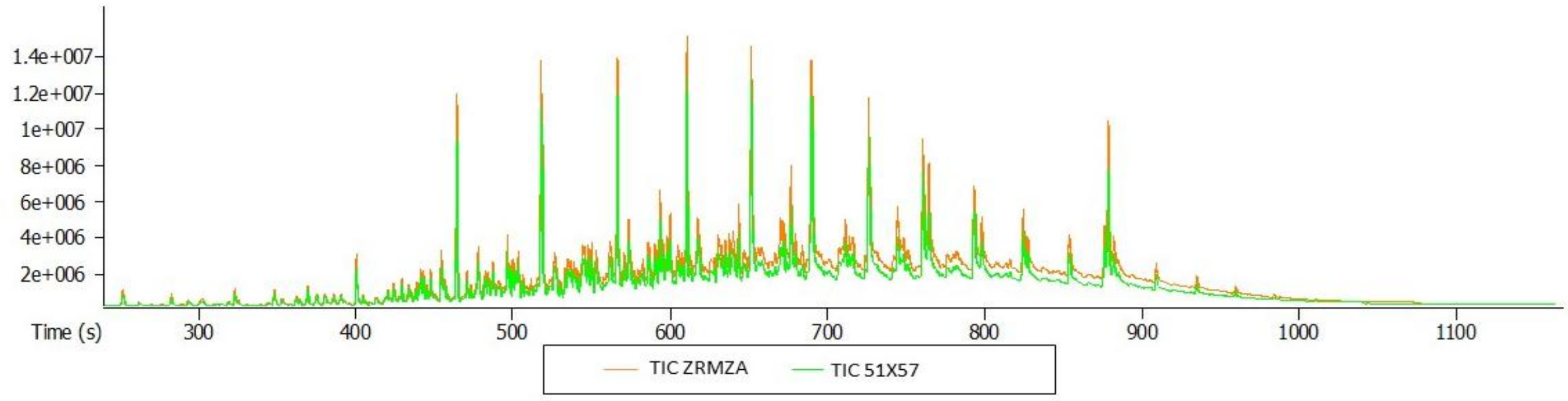

Supplementary Figure S2. 1D GC-TOF/MS chromatograms (TIC) for a representative sample (ZRMZA) and the outlier sample (51X57).

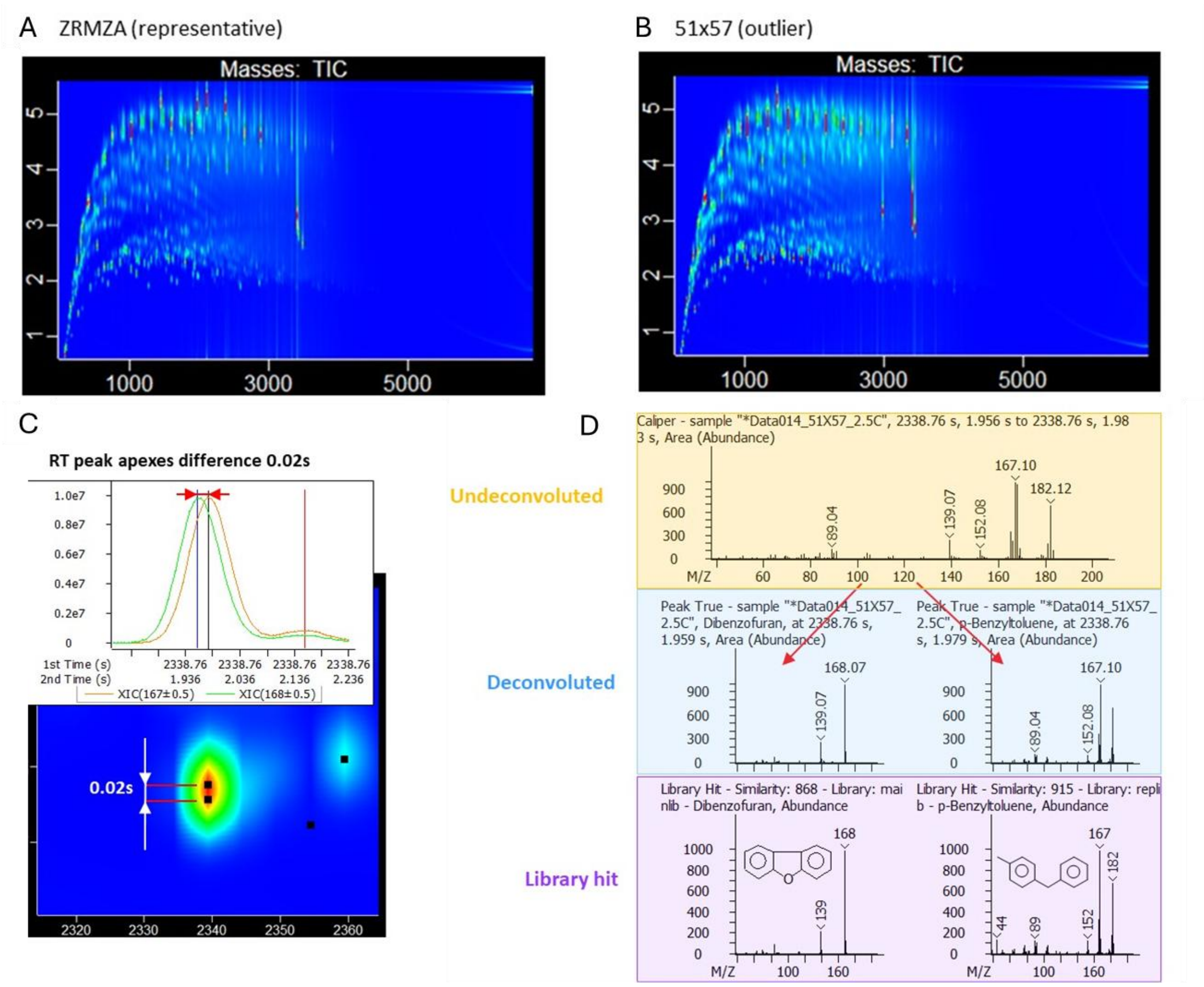

Supplementary Figure S3. GC×GC-TOF/MS TIC contour plots for a representative sample (ZRMZA; A) and the outlier sample (51X57; B), together with an example of a partially overlapping feature pair in sample 51X57 (C) and corresponding EI mass spectra/library-match comparison before and after deconvolution (D). The figure illustrates how comprehensive separation and spectral deconvolution reduce mixed-spectrum effects in congested chromatographic regions and support more defensible tentative library matching.
